# Taphonomic experiments imply a possible link between the evolution of multicellularity and the fossilization potential of soft‐bodied organisms

**DOI:** 10.1002/ece3.7120

**Published:** 2020-12-15

**Authors:** Elena Naimark, Dmitry Kirpotin, Natalia Boeva, Vladimir Gmoshinskiy, Maria Kalinina, Yulia Lyupina, Alexander Markov, Michail Nikitin, Alexander Shokurov, Dmitry Volkov

**Affiliations:** ^1^ Borissiak Paleontological Institute Russian Academy of Sciences Moscow Russia; ^2^ Kirpotin Biotechnology Consulting San Francisco CA USA; ^3^ Institute of Geology of Ore Deposits, Petrography, Mineralogy, and Geochemistry Russian Academy of Sciences Moscow Russia; ^4^ Faculty of Biology Moscow State University Moscow Russia; ^5^ Frumkin Institute of Physical Chemistry and Electrochemistry Russian Academy of Sciences Moscow Russia; ^6^ Koltzov Institute of Developmental Biology Russian Academy of Sciences Moscow Russia; ^7^ Belozersky Institute for Physico‐Chemical Biology Moscow State University Moscow Russia; ^8^ Kharkevich Institute for Information Transmission Problems Russian Academy of Sciences Moscow Russia; ^9^ Institute of Biology of the Southern Seas Russian Academy of Sciences Moscow Russia; ^10^ Faculty of Chemistry Moscow State University Moscow Russia

**Keywords:** cell adhesion molecules, fossilization, metazoa, multicellularity, sediment, soft‐bodied fossils

## Abstract

The reliability of evolutionary reconstructions based on the fossil record critically depends on our knowledge of the factors affecting the fossilization of soft‐bodied organisms. Despite considerable research effort, these factors are still poorly understood. In order to elucidate the main prerequisites for the preservation of soft‐bodied organisms, we conducted long‐term (1–5 years) taphonomic experiments with the model crustacean *Artemia salina* buried in five different sediments. The subsequent analysis of the carcasses and sediments revealed that, in our experimental settings, better preservation was associated with the fast deposition of aluminum and silicon on organic tissues. Other elements such as calcium, magnesium, and iron, which can also accumulate quickly on the carcasses, appear to be much less efficient in preventing decay. Next, we asked if the carcasses of uni‐ and multicellular organisms differ in their ability to accumulate aluminum ions on their surface. The experiments with the flagellate *Euglena gracilis* and the sponge *Spongilla lacustris* showed that aluminum ions are more readily deposited onto a multicellular body. This was further confirmed by the experiments with uni‐ and multicellular stages of the social ameba *Dictyostelium discoideum*. The results lead us to speculate that the evolution of cell adhesion molecules, which provide efficient cell–cell and cell–substrate binding, probably can explain the rich fossil record of soft‐bodied animals, the comparatively poor fossil record of nonskeletal unicellular eukaryotes, and the explosive emergence of the Cambrian diversity of soft‐bodied fossils.

## Graphical abstract

We present the results of long‐term (1–5 years) taphonomic experiments with *Artemia salina* buried in different sediments. The results imply that fossil preservation of soft‐bodied organisms depends critically on fast deposition of Al and Si ions and that the evolution of cell adhesion molecules in the early multicellular organisms probably played an important role in the sudden appearance of diverse fossil record of multicellular soft‐bodied organisms around the Precambrian/Cambrian boundary.

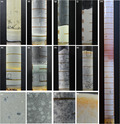

## 1 INTRODUCTION

How can a soft‐bodied organism escape decomposition and turn into a fossil? This question is crucial for understanding the patterns in the fossil record, such as the fast growth of the observed diversity of metazoans during the Cambrian including soft‐bodied organisms (sedimentary deposits with fossilized soft‐bodied organisms or soft tissues are known as Lagerstätten). Many hypotheses have been suggested to explain the fossilization of soft‐bodied organisms (SBO), as well as different modes of their preservation, by various abiotic and biotic factors. Abiotic factors that presumably enhance SBO preservation include low oxygenation (Allison, 1988; McCoy et al., 2015a,b; Naimark, Kalinina, Shokurov, Boeva, et al., 2016; Naimark, Kalinina, Shokurov, Markov, et al., 2016), low or high acidity induced by decay (Allison, 1988; Berner, 1968; Briggs & Kear, 1997; Naimark, Kalinina, Shokurov, Boeva, et al., 2016; Sagemann et al., 1999; Wilson & Butterfield, 2014), deposition of iron (Petrovich, 2001; Schiffbauer et al., 2014), phosphorus (Raff et al., 2008), calcium (Butterfield, 2003), silicon (Strang et al., 2016), or "tanning" by aluminum (Naimark, Boeva, et al., 2018; Naimark, Kalinina, Shokurov, Boeva, et al., 2016; Naimark, Kalinina, Shokurov, Markov, et al., 2016; Wilson & Butterfield, 2014). Biotic factors include the lack of bioturbation and detritus consumers (Garson et al., 2011; but see: Pratt & Kimmig, 2019), the slow proliferation of sulfate reducers due to the depletion of sulfur and iron caused by early surface cementation (Gaines et al., 2012; Hammarlund et al., 2011; McCoy et al., 2015b), inhibition of bacterial growth and absorption of bacterial lytic enzymes by some clays (Butterfield, 1995; Kompantseva et al., 2011; McMahon et al., 2016), or, otherwise, increased activity of cyanobacteria (or other bacteria) producing a mineral “death mask” (Darroch et al., 2012; Gehling, 1999; Martin et al., 2004; Raff et al., 2013). Such a wide variety of hypotheses reflects the fact that SBO can be fossilized in many different paleoenvironments, and many environmental factors can possibly affect the fossilization potential of SBO. However, we believe that there should also be some general mechanisms for the preservation and fossilization of SBO, since animals with very different structures and biochemical compositions are typically preserved in the same locality (Fu et al., 2019). If so, experiments with even a single model animal can help identify these common mechanisms.

We asked (a) whether there are some core processes among this wide variety that underpin the preservation of SBO, and (b) whether these processes differ between uni‐ and multicellular organisms. The latter question stems from the fact that the Ediacaran and Phanerozoic fossil record of multicellular SBO is quite rich and diverse (Muscente et al., 2017), whereas unicellular organisms are widely believed to leave no fossil record, except for those with hard parts (cysts of green algae, mineral endoskeletons of Radiolaria, tests of testate amoebae, etc.) (Lahr et al., 2019).

In order to identify core chemical pathways in SBO fossilization, we conducted a line of long‐term taphonomic experiments (1.5–5 years: the longest of this type) with the model multicellular organism *Artemia salina* buried in different sediments. Some results were published previously (Naimark, Boeva, et al., 2018; Naimark, Kalinina, & Boeva, 2018; Naimark, Kalinina, Shokurov, Boeva, et al., 2016; Naimark, Kalinina, Shokurov, Markov, et al., 2016; Naimark, Kalinina, Shokurov, et al., 2018). They are combined with the new ones presented here (Table 1). The results reveal coordinated changes in pH and mineral composition of the sediments, as well as in the elemental and chemical composition of the carcasses. These changes appear to be different facets of the complex preservational process in sediment. Overall, the results imply that the early deposition of aluminum and/or silica ions on decaying tissues significantly enhances SBO preservation.

**TABLE 1:** Taphonomic experiments with *Artemia salina* buried in different sediments

Sediment	Duration	Task	Source
Mg‐chlorite (clinochlore) in ASW^a^	12 months	To test decay rate, mineralogical transformations of the sediment, elemental composition and biochemical profile of the carcasses to infer components favouring preservation; to assess the influence of iron, magnesium, and calcium	Naimark, Kalinina, Shokurov et al. (2018)
Fe‐chlorite (chamosite) in ASW	12 months		This study
Kaolinite in ASW	12 months	To test decay rate, mineralogical transformations, composition of the carcasses; influence of aluminum and calcium	Naimark, Boeva, et al. (2018)
Artificial SiO_2_ in ASW	12 months	To test decay rate, composition of the carcasses; influence of silicon	This study
Montmorillonite in fresh water	5 years	To test decay rate, mineralogical transformations, composition of the carcasses; influence of iron and silicon	This study
Sediment‐free control in ASW	12 months	To test decay rate and composition of the carcasses with and without sediment; influence of calcium	Naimark, Kalinina, Shokurov, Markov et al. (2016); Naimark, Boeva et al. (2018); Naimark, Kalinina, Shokurov et al. (2018)

^a^ ASW—artificial sea water (see Section 2 for its chemical composition).

Based on these results, we asked if the early deposition of aluminum proceeds differently on the surface of single‐ and multicellular organisms. We used the flagellate (Excavata) *Euglena gracilis* and the sponge *Spongilla lacustris* as model systems in these experiments. We also experimented with the unicellular and multicellular stages of the colonial amoebae *Dictyostelium discoideum*. In all cases, we found enhanced deposition of aluminum ions in multicellular organisms/stages compared to unicellular ones. It is noteworthy that the transition from unicellular to multicellular stage in *D. discoideum* is accompanied by the appearance of “intercellular glue,” that is, by the expression of cell adhesion molecules (CAMs). The results led us to speculate that the emergence of CAMs at the early stages of the evolution of multicellularity might explain why the fossil records of unicellular and multicellular soft‐bodied organisms are so dramatically different in richness.

## 2 MATERIALS AND METHODS

### 2.1 Burial experiments

#### 2.1.1 Sediments used in taphonomic experiments with *Artemia salina*

We buried *A. salina* in five different fine‐grained sediments (four clays and artificial silica) in order to assess the relative importance of different components and properties of the sediment for the preservation of SBO. Sediments of similar mineralogy are common in Lagerstätten (Anderson et al., 2018). However, we made no attempt to model real sediment types involved in Lagerstätten formation; rather, we sought to better understand the general principles of SBO fossilization. The main criterion for choosing sediments was the purity of the available samples which was crucial for the subsequent analysis and interpretation.

The initial chemical composition of the sediments, measured by X‐ray fluorescent analysis, is shown in Table 2. The kaolin came from the Polog deposit (Ukraine) and contained 97% of kaolinite in the purified sample (see Naimark, Kalinina, Shokurov, Markov, et al., 2016 for details). The clinochlore originated from the Akhmetievskoe deposit (Ural mountains) and was given to us from the collection of Fersman Mineralogical Museum (Moscow). The chamosite originated from the Kursk Magnetic Anomaly, the collection stored in the museum of the Institute of Geology of Ore Deposits, Petrography, Mineralogy and Geochemistry (Moscow). The montmorillonite was from Tikhmenievskoe deposit (Sakhalin), from the collection of one of the authors (N.B.). Artificial silica was precipitated from perlite by thermal decomposition (150°C) with NaOH and consequent neutralization by sulfuric acid yielding porous, amorphous siliceous particles. We did not use glass beads or sand as a model for siliceous sediment (like in Newman et al., 2019) because glass beads and sand are chemically highly inert.

**TABLE 2:** Initial chemical composition of the experimental sediments (measured by X‐ray fluorescent analysis; calculated as oxides wt%)

Elements	CO_2_ (%)	Na_2_O (%)	MgO (%)	Al_2_O_3_ (%)	SiO_2_ (%)	K_2_O (%)	CaO (%)	TiO_2_ (%)	MnO (%)	Fe_2_O_3_ (%)	P_2_O_5_ (%)	S (%)
Sample name												
Montmorillonite	11.0	1.35	1.96	10.52	71.65	0.25	0.59	0.1	0.06	1.96	0.01	<0.02
Kaolinite	13.95	<0.1	0.21	36.83	45.81	0.49	0.35	0.84	<0.01	1.31	0.03	<0.1
Artificial SiO_2_	10.3	0.7	0.01	0,01	88.92	0.06	0.01	0	0	0.01	0	0
Chamosite	9.99	0.04	1.13	17.43	23.40	0.01	0.40	9.95	0.15	37.12	0.18	0.07
Clinochlore	12.11	<0.02	30.33	20.74	27.03	<0.02	0.22	0.59	0.104	8.63	0.16	0.05
ASW	0	24	7.19	0.02	0.02	3.80	2.71	0	1.60	0	0	0.05

Nonsterile artificial sea water (ASW; brand “Tetra”) was prepared according to the instructions for this brand. We checked its pH (7.4–7.8) and salinity (24‰); the chemical composition was assessed by X‐ray fluorescent analysis (Table 2). For the freshwater experiment with montmorillonite, we took tap water and kept it open under nonsterile conditions in a glass jar for a week to obtain a stable microbial consortium.

To prepare the sediments, all samples of minerals were ground finely in an agate mortar. Next, they were mixed in settled tap water and equilibrated for half a minute, allowing large and/or heavy particles to gravitate to the bottom. Then, the upper portion (approximately 2/3 of the suspension) was decanted. This upper portion was dried, ground again, and mixed in ASW (or tap water in the case of montmorillonite) in proportion of 3 g/100 ml. The resulting suspensions were actively mixed with bubbling from an air pump for 48 h to remove clay pellets.

#### 2.1.2 Model organism

We used nauplii L1 and L2 of *Artemia salina* (Branchiopoda, Crustacea) for the burial experiments. This model organism is suitable for our purposes because small crustacean fossils are found in many Lagerstätten, and also because its bright orange coloration helps to find the carcasses in the sediment. The nauplii were obtained from eggs after 36–48 h of incubation and killed by keeping in fresh water for 4–6 h.

#### 2.1.3 Design of the taphonomic (burial) experiments

Our taphonomic experiments (Table 1) imitate rapid burial of SBO under a sediment layer, which is believed to be a common condition for Lagerstätten formation. The experimental design was described in our earlier works (Naimark, Boeva, et al., 2018; Naimark, Kalinina, Shokurov, Boeva, et al., 2016; Naimark, Kalinina, Shokurov, Markov, et al., 2016; Naimark, Kalinina, Shokurov, et al., 2018). The homogeneously mixed clay suspensions were poured into high glass tubes (50–70 cm in height, 1.0–1.5 cm in diameter; as we observed in multiple experiments, this variation did not affect preservation). A total of 200–300 mg of dead nauplii of *Artemia salina* was put on top of the suspension in each tube, and then, the carcasses and mineral particles were allowed to settle together. The nauplii accumulated in the middle or in the upper part of the kaolinite sediment. In silica, the nauplii sank approximately with the same speed as the smallest particles, so the carcasses were deposited within the thin top layer of the sediment. In clinochlore and chamosite, the particles sank faster than the dead nauplii, so the majority of the nauplii were concentrated in the topmost layer and at the surface of the sediment. To cover them on top, we added a portion of the respective sediment (clinochlore or chamosite, according to the type of experiment). Montmorillonite formed a thin colloidal solution which was gradually settling down throughout the 5‐year experiment. Carcasses were distributed in the middle and lower parts of the colloid; their concentration gradually increased from top to bottom. The bright orange coloration of the nauplii was clearly visible in all sediments. We used the same sediments without nauplii as controls; there was also a sediment‐free control (nauplii in the ASW).

All tubes were sealed by one layer of Parafilm, which decreases evaporation and gas exchange but does not block these processes completely. Then, the tubes stayed undisturbed in the dark at room temperature (25–28°C). After 1 year (5 years for the montmorillonite), they were opened and analyzed.

There were 15 replicate tubes for the kaolinite, which were used to demonstrate the robustness of the decay pattern. We observed no difference in the pattern of decay between the replicate tubes (Naimark, Boeva, et al., 2018). There were also three replicate experiments (tubes) for the clinochlore, and two for the clinochlore control and the chamosite. Other experiments had no replication. However, each tube had multiple carcasses, so the lack of replicate tubes does not indicate a lack of replicate decaying carcasses. All replications demonstrated the same decay pattern and very similar pH changes and degree of preservation of the carcasses (Naimark, Boeva, et al., 2018; Naimark, Kalinina, Shokurov, et al., 2018).

#### 2.1.4 Degree of preservation of *A. salina* nauplii

Small portions of the sediment with carcasses were put in the Petri dish, diluted with ASW, and investigated under optical microscope (Zeiss Stemi SV11) to count the nauplii and assess the degree of their preservation. We categorized the remains in five groups according to the presence of morphologically recognizable limbs and gut and the general body shape. The procedure and the characteristics of the five preservational groups are described in detail in Naimark, Kalinina, and Boeva (2018).

Group 1 comprised carcasses with all limbs, gut and body shape well preserved. Specimens in group 2 had well‐preserved limbs and body shape but no traces of the gut. Unlike freshly killed specimens or specimens from other preservational groups, the group 2 specimens were rigid and did not tend to fold in wrinkles when placed on a hard surface. Group 2 was rare in sediment‐free controls but common in sediments. Groups 3–5 comprised specimens with preservation gradually deteriorating from only moderately damaged cuticle, gut and limbs in group 3 to separate limbs or limbless bodies in group 5 (Naimark, Kalinina, & Boeva, 2018). The overall degree of preservation was estimated as the ratio of the well‐preserved groups 1 and 2 to the poorly preserved groups 3, 4, and 5.

#### 2.1.5 pH measurements

To measure pH, 0.5–1 ml samples were taken by a pipette from the water column 10–15 cm above the sediment, from the water–sediment interface (including 1–2 mm of water above the sediment and the uppermost sediment layer), and from the middle of the sediment. For the montmorillonite variant, samples were taken 5 cm under the water surface, 1 cm above the red spot (see below), in the middle of the red spot, 1 cm under the red spot, and 1 cm above the bottom of the tube.

All pH measurements were performed within 2 days after opening the tubes; during this time the samples were stored at +4°C. Liquid samples were tested according to the standard protocol using a combination glass pH electrode, calibrated in a standard buffered solution. Portions of the sediment were stirred using a vortex type stirrer without preliminary filtration until a uniform suspension was formed, and then, the pH was measured with the same pH meter 3–5 times for each sample (the results represent an average of these measurements).

#### 2.1.6 Mineralogical analyses

Samples for mineralogical analysis were taken from the middle of the sediments (where numerous carcasses were buried), or from the red layer (see below) in the case of montmorillonite. Sediment samples were investigated by Simultaneous Thermal Analysis (STA) that combines differential scanning calorimetry (DCA) and thermogravimetry (TG). TG/DCA curves were recorded with the STA equipment (NETZSCH STA 449 F3 Jupiter®) at a heating rate of 10°C/min at room atmosphere. Mineral transformations caused by organic decay in kaolinite and clinochlore were described earlier (Naimark, Boeva, et al., 2018; Naimark, Kalinina, Shokurov, et al., 2018). Here, we report the transformations in montmorillonite and chamosite in comparison with correspondent controls without nauplii.

#### 2.1.7 Elemental composition of the exhumed nauplii

Fine anatomical structures and elemental composition of the carcasses were studied under SEM (Zeiss EVO‐50) and associated SEM⁄EDX (energy dispersive X‐ray spectroscopy INCA Oxford 350). To prepare the samples for SEM analysis, the nauplii were rinsed of salts and sediment particles, as described in Naimark, Kalinina, and Boeva (2018). SEM imaging was performed on glass supports, and SEM⁄EDX point analyses on copper supports to allow for measurement of Al, Si, and Fe. We analyzed separately the adhered mineral particles and the surface tissues of the carcasses (free of bacteria and visible adhered mineral particles). This allowed us to distinguish between the tissue intake of dissolved elements and mere adhesion of minerals.

#### 2.1.8 Molecular content of the exhumed nauplii: FT‐IR spectroscopy

The exhumed carcasses were tested for the molecular content in a Fourier‐transform infrared (FT‐IR) spectrometer Vertex 70 (Bruker Optik GmbH) with a GladiATR attenuated total reflection (ATR) attachment with a diamond crystal (Pike Technologies). This equipment makes it possible only to measure bulk biochemical content of 3–5 specimens together, but not separately. The range of spectra registration was 6000–350 cm^−1^ with the resolution 2 cm^−1^, number of scans—256. A total of 10–15 carcasses were rinsed in distilled water and then transferred on the crystal and dried directly on it. The spectrum was registered after complete drying. Sediment particles from the experimental tubes (*Artemia* with kaolinite, montmorillonite, silica, clinochlore, and chamosite) were also washed in distilled water and dried in the same way as the carcasses; their spectra were compared with the corresponding carcasses. We also compared the spectra of the carcasses from the sediments, from sediment‐free control, and freshly killed nauplii prepared in the same way. To estimate the initial adherence of sediment particles to the nauplii, we used freshly killed nauplii incubated in kaolinite for 1 day and then rinsed and dried. To investigate the difference between well and poorly preserved carcasses, we applied differential FT‐IR testing to the representatives of preservational groups 1–2 and 5 from kaolinite.

### 2.2 Deposition of aluminum on single‐celled and multicellular bodies

#### 2.2.1 Model organisms

For these experiments, we used unicellular and primitive multicellular model organisms: freshwater sponge *Spongilla lacustris* (Demospongiae, Porifera), flagellate *Euglena gracilis* (Euglenozoa, Excavata), and social ameba *Dictyostelium discoideum* (Amoebozoa). While the two former organisms are different in all biochemical aspects, *D. discoideum* has both unicellular and multicellular stages. Under certain environmental conditions, unicellular amoebae gather to form a loose multicellular aggregate. This then transforms into a tight aggregate (mound), then into a motile “slug,” a culminant, and finally a well‐differentiated spore‐producing fruiting body. Thus, in *D. discoideum*, we have a convenient example of single‐celled and multicellular organisms with essentially the same biochemistry except for a set of CAMs and ECMs which is expressed predominantly at multicellular stages (these molecules are essential for cell‐to‐cell adhesion). Importantly, although CAMs are not identical in *D. discoideum* and animals, their biochemical properties and functions are quite similar, and cell adhesion mechanisms employed during *D. discoideum* morphogenesis are also strikingly similar to animal cell adhesion (Abedin & King, 2010).

The first two organisms are freshwater, and the third one is terrestrial. This difference can be a source of an additional variability. However, the multicellular forms possess the CAMs while the unicells do not irrespective of their environment. According to the logic of our study, in this experiment we could use any multicellular and unicellular organisms which do not have skeletons, are robust to mechanical and chemical manipulations, and their autofluorescence and fluorescence after lumogallion staining differ from lumogallion‐Al fluorescence (see below).

*Spongilla lacustris* was collected in summer 2018 from Moskva river (Zvenigorodskaya biological station, Russia) and kept as a cryopreserved viable specimen.

*Euglena gracilis* was cultured at the Faculty of Biology, Moscow State University, on the Kramer‐Mayer medium (1 g/L (NH_4_)_2_HPO_4_, 1 g/L KH_2_PO_4_, 0.8 g/L Na_2_C_6_H_5_O_7_ × 5H_2_O, 0.2 g/L MgSO_4_, 0.02 g/L CaCl_2_, 3 mg/L Fe_2_(SO_4_)3 × H_2_O, 1.8 mg/L MnCl_2_ × 4H_2_O, 1.3 mg/L CoCl_2_ × 6H_2_O, 0.4 mg/L ZnSO_4_ × 7H_2_O, 0.2 mg/L Na_3_Mo_4_ × 2H_2_O, 0.02 mg/L CuSO_4_ × 5H_2_O, 20 μg/L thiamine, 10 μg/L cobalamine, ethanol to 0.2 M; pH 6.6–6.7).

*Dictyostelium discoideum* strain DBS0237637 from Dicty Stock Center (Northwestern University, Chicago, IL, USA) was grown on *E. coli* lawn on agar plates with SM5 medium (2 g/L glucose, 2 g/L Bacto Peptone (Oxoid), 2 g/L yeast extract (Oxoid), 0.2 g/L MgCl_2_, 1.9 g/L KHPO_4_, 1 g/L K_2_HPO_4_, 15 g/L agar).

Before the experiment, *Spongilla* and *Euglena* were killed by freezing in liquid nitrogen, and *D. discoideum* by keeping in 500 µM/L sodium azide for 15 min (Sadiq, 1995).

#### 2.2.2 Deposition of aluminum on *Spongilla lacustris*, *Euglena gracilis,* and *Dictyostelium discoideum* studied by lumogallion staining

Dead *E. gracilis* cells were transferred to glass slides and dried briefly to adhere to the glass surface. *S. lacustris* were processed in small Petri dishes. The culture of *D. discoideum* with unicellular amoebae and different multicellular stages (mounds, sorogens, and fruiting bodies) was washed off from the agar plate with distilled water and transferred onto glass chamber slides and left to air dry briefly to adhere to the bottom of the glass chamber. Then, sodium azide was removed by rinsing with distilled water.

Treatment with aluminum was carried out by incubation with 1 mM solution of alum (KAl(SO_4_)_2_) in distilled water at room temperature for 30 min. To remove unbound aluminum, the specimens on the glass slides or in Petri dishes were rinsed 5 times with distilled water, then fixed in 2% paraformaldehyde (PFA) for 20 min, and rinsed 2 times with distilled water to remove PFA.

#### 2.2.3 Detecting Al deposited on the surface of cells

The presence of bound aluminum was detected with standard lumogallion (5‐chloro‐3[(2,4‐dihydroxyphenyl)azo]‐2‐hydroxybenzenesulfonic acid) staining, described elsewhere and used to detect aluminum at low (around 2 μM) concentrations (Kataoka et al., 1997; Mold et al., 2014). Lumogallion is a very sensitive dye for bound aluminum detection. Lumogallion‐Al complex has strong fluorescence at 488 nm (green); thus, the intensity of green fluorescence allows for the comparison of the amount of bound aluminum between the samples. The controls were represented by Al‐negative variant (a carcass incubated in lumogallion without Al to reveal tissue‐specific lumogallion binding), and autofluorescence control (a carcass without Al and without lumogallion).

The samples were stained in 0.025 mM lumogallion in 0.1 M sodium acetate buffer pH 5.2 at 50°C for 50 min in the dark. The lumogallion solution was removed, and the stained preparations were rinsed with distilled water.

In the case of *D. discoideum,* samples were also counterstained with the DNA dye Hoechst 342 in a conventional manner to visualize the positions of cell nuclei.

Prepared samples covered with a cover glass were immediately examined under a Leica DMR Fluorescent Microscope with an UV excitation and emission at 488 nm (green, characteristic for lumogallion‐Al complex); colonial amoebae were also examined at 408 nm (blue, Hoechst–DNA). *E. gracilis* preparations were also examined under the confocal fluorescent microscope Leica TCS SP5 at UV excitation and 488 nm emission to reveal cellular localization of the fluorescent structures. Each sample was prepared in triplicate. All images for each model organism and their controls were made at the same exposition time according to the standard protocols for fluorescence studies. This allowed us to visually compare the fluorescence intensity between objects. As the differences were quite prominent and similar between replicates, we did not attempt to obtain quantitative measures of fluorescence intensity.

## 3 RESULTS

### 3.1 Burial experiments

#### 3.1.1 The pattern of decay of the model soft‐bodied organism *Artemia salina* in different sediments

**FIGURE 1:**
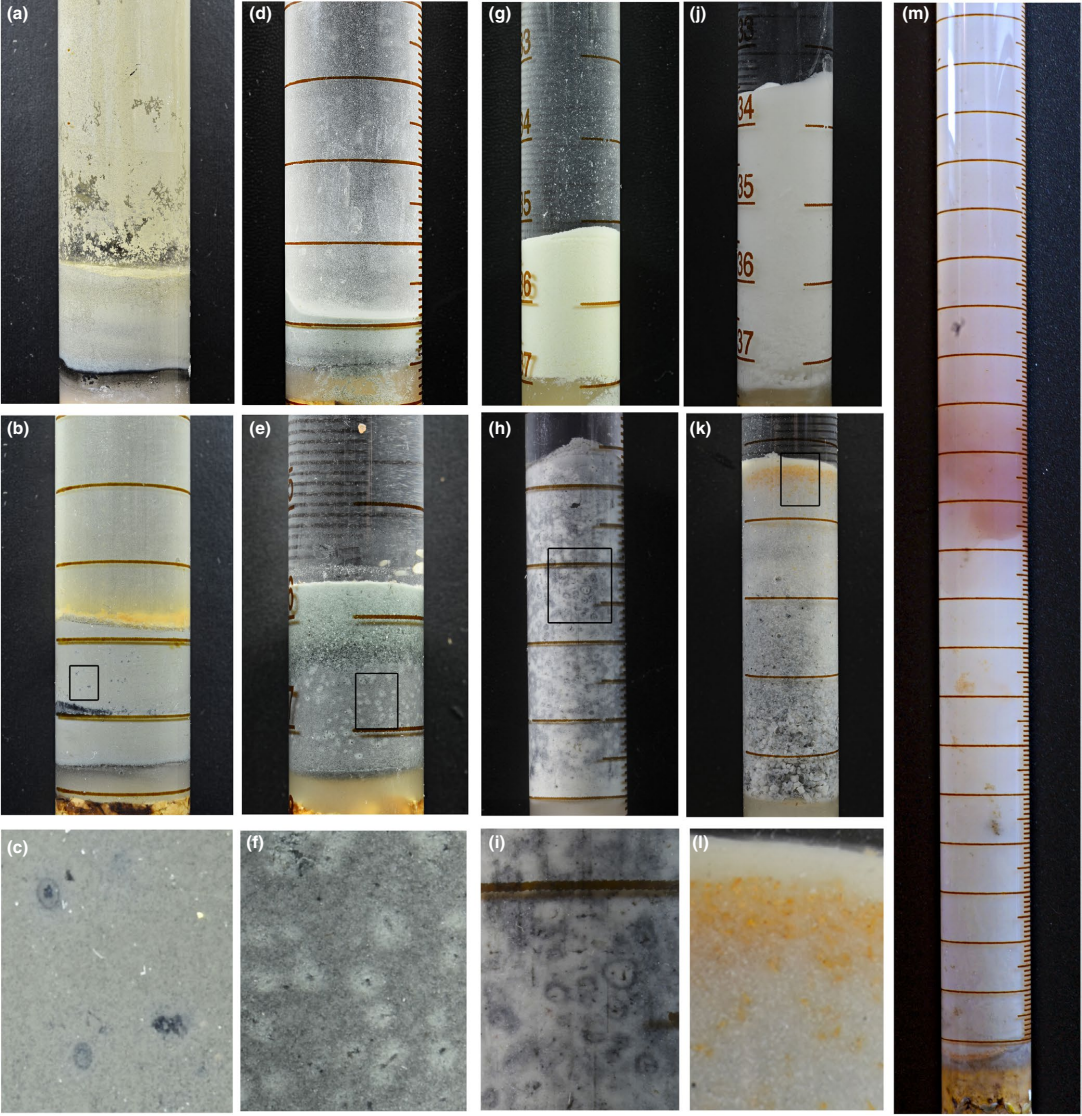
Taphonomic (burial) experiments: experimental and control tubes with sediments and buried *Artemia salina*. (a–c)—chamosite: a, control without *A. salina*; b, sediment with *A. salina* after 12 months. The black layer contains numerous carcasses; the orange top layer is comprised of carcasses covered by some iron species. (c) enlarged area (framed in b) showing spots around nauplii. (d–f)—clinochlore: the same as the previous triplet; multiple light spots around the carcasses are well visible. (g–i)—kaolinite: complex light spots around the carcasses are visible. (j–l)—artificial SiO_2_; orange coloration marks the layer in which the carcasses accumulated. (m)—montmorillonite after 5 months. The red layer is well visible; orange and brownish spots (below the red layer) and the orange layer at the bottom are carcasses

In our burial experiments, we used five different model sediments with various mineral and chemical compositions: chamosite, clinochlore, kaolinite, montmorillonite, and artificial silica (Table 2, see Section 2). The experimental aqueous sediments with the buried nauplii of *A. salina* (see Section 2) quickly (within the first week) changed their coloration (Figure 1). In the kaolinite, clinochlore, and chamosite, prominent light‐colored spots formed around every carcass (Figure 1c,f,i). In the silica, dead nauplii clustered in a subsurface layer which remained light‐colored on the otherwise darkened background (Figure 1k,l). In the montmorillonite, a bright red layer formed by the 5th month and stayed well visible to the end of the experiment (5 years, Figure 1m). The patterns in the sediment controls (prepared by the same protocol but without any buried *Artemia*) differed dramatically: their coloration remained unchanged (Figure 1a,d,g,j). This difference implies some active chemical, organic–mineral processes within the sediments that contain decaying soft‐bodied organisms.

#### 3.1.2 The degree of preservation of the model SBO in different sediments

In order to quantitatively assess the degree of preservation of the buried nauplii, we assigned them to five preservational groups which reflect successive stages of decomposition (see Section 2 for details).

**FIGURE 2:**
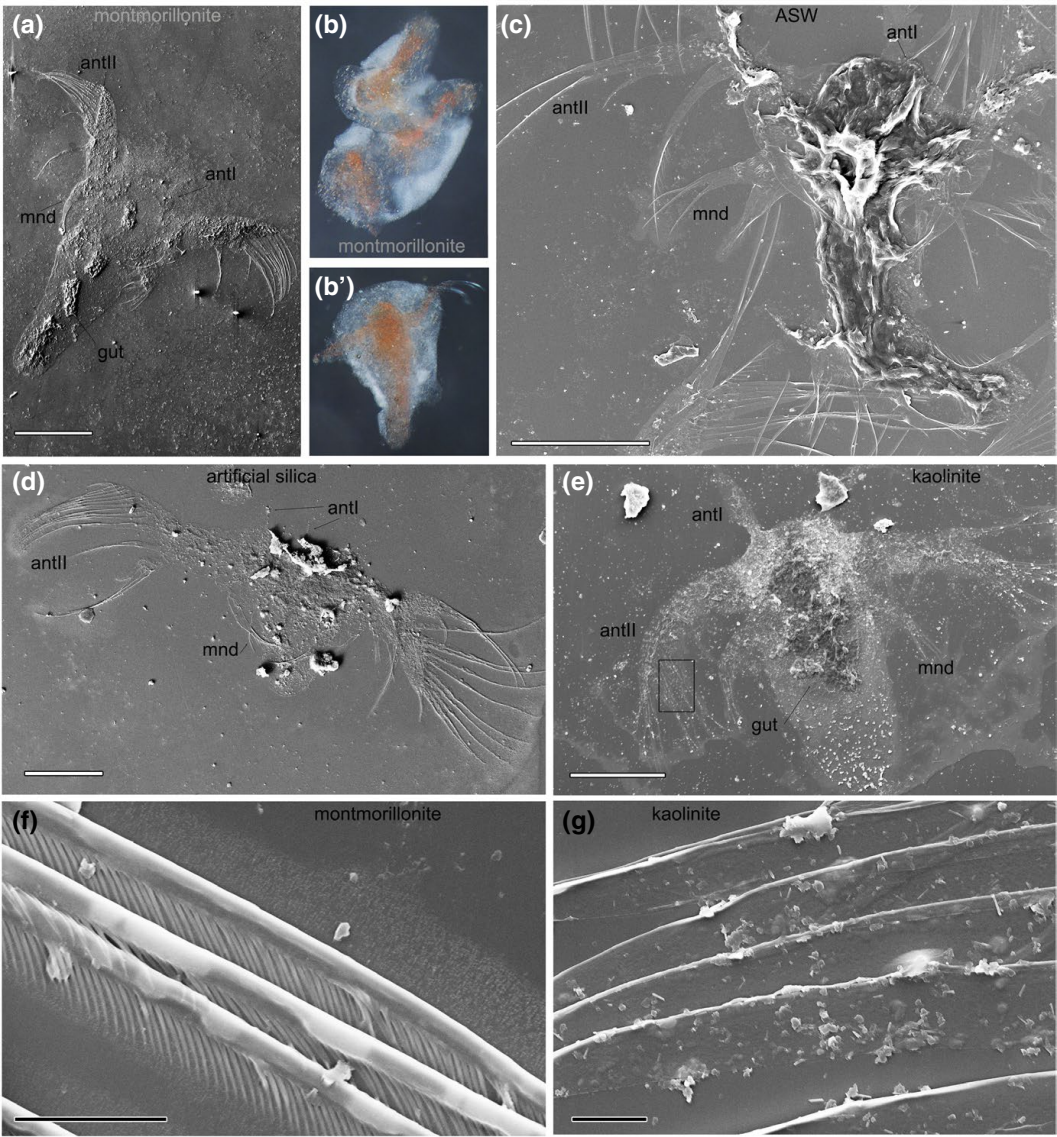
Nauplii of *Artemia salina* from different sediments (SEM images, except b). (a) Well‐preserved specimen with limbs and gut (preservational group 1) from the montmorillonite. (b, b′): Specimens from the montmorillonite that remained wrapped in clay envelopes after rinsing. (c) Nauplius from the ASW control with poorly preserved, shapeless body (preservational group 3). (d) Nauplius from artificial silica, preservational group 2 (overall body shape and limbs preserved, no internal anatomy). (e) Group 1 specimen from the kaolinite. (f) Perfectly preserved filter apparatus on antenna II of a specimen from the montmorillonite. (g) The bases of antenna II chaetae with tightly adhered small mineral particles (magnified framed area from (e), rotated 90° clockwise). antI, antII—antennae I and II; mnd—mandibulae. Scale bars 200 μm (a–e), 3 μm (f, g)

**FIGURE 3:**
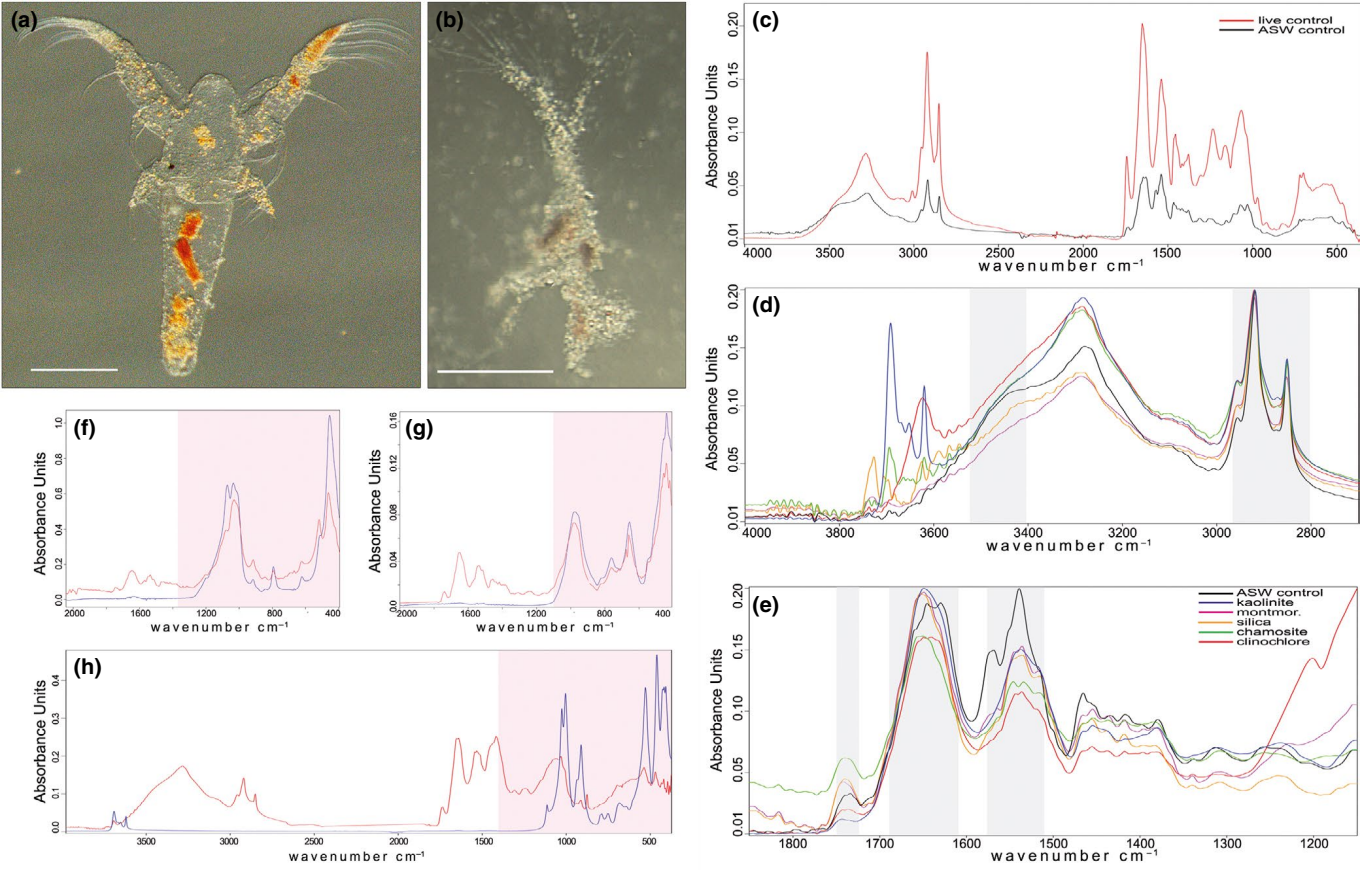
Biochemical composition of the carcasses: FT‐IR spectra for specimens from the sediments and controls. (a) An example of a well‐preserved specimen (group 1; buried in montmorillonite for 5 years; scale bar 100 μm). (b) An example of a poorly preserved specimen (group 4; buried for 1 year in kaolinite; scale bar 100 μm). (c) FT‐IR of the live control (freshly killed nauplii) and the ASW control. (d) Wavelength interval containing chitin characteristic bands (gray shadow) in carcasses from all sediments. (e) Wavelength interval containing proteins characteristic bands (gray shadow) in carcasses from all sediments. Vertical axes in (d) and (e) are normalized to intensities. (f) Montmorillonite‐hosted carcasses (5 years) and corresponding sediment particles; wavelength interval characteristic for the montmorillonite shown in pink; (g) The same for the clinochlore; wavelength interval characteristic for the clinochlore shown in pink. In (f) and (g), red lines are carcasses spectra, and blue lines are sediment spectra. (h) One‐day control in kaolinite (red line) and the corresponding kaolinite particles (blue line); wavelength interval characteristic for the kaolinite shown in pink

The best preserved carcasses (group 1) from the montmorillonite, kaolinite, and silica retained very fine external anatomical structures such as the filter apparatus on antennae 2 and the chaetae (Figure 2f,g). They also preserved the naturally shaped remains of the gut (Figures 2a,e and 3a), which usually rapidly dissolves when buried in liquid media without sediment (Butler et al., 2015). We never observed such fine anatomical details in the representatives of groups 3, 4 and 5.

Group 2 was comprised of well‐shaped rigid external integument with finely preserved limbs but completely decayed internal anatomy (Figure 2d). This group is specific to decay in sediment as it was abundant in all sediments but almost absent in the sediment‐free control (Table 3; Butler et al., 2015; Naimark, Kalinina, Shokurov, et al., 2018).

**TABLE 3:** Degree of preservation of *Artemia salina* nauplii in different sediments: average percentages of preservational groups 1–5 and standard deviations in parentheses

	Group 1	Group 2	Group 3	Group 4	Group 5	Number of samples (total number of specimens in all samples)	Overall degree of preservation: (gr1 + gr2)/(gr3 + gr4+gr5)
	All limbs, gut and body shape well preserved	All limbs and body shape well preserved, no traces of the gut	Moderately damaged cuticle, gut, and limbs	Body shape and limbs only partially preserved	Separate limbs or bodies without limbs		
Montmorillonite^a^	20.3	50	3.7	7.4	18.5	1 (54)	2.4
Kaolinite	14.7 (3.1)	40.8 (6.1)	11.3 (0.1)	13.6 (4.7)	19.4 (4.6)	3 (281)	1.25
SiO2	30.75 (1.6)	14.3 (3.3)	15.1 (2.0)	12.1 (2.0)	27.1 (3.1)	3 (278)	0.82
chamosite	14.8 (2.8)	14.6 (1.4)	22.4 (8.7)	25.9 (1.3)	22 (7.9)	3 (113)	0.42
clinochlore	5 (4.8)	16.5 (6.3)	15.6 (1.4)	22.1 (6.7)	40 (5.9)	5 (532)	0.27
ASW^a^	6.3	1.25	11.2	23.8	57.5	1 (80)	0.08

^a^ Only one sample was studied, and so there are no standard deviations.

The montmorillonite experiment continued for 5 years, and we expected the majority of the remains to be in groups 4 and 5. Contrary to our expectations, the carcasses from this five‐year‐old sediment were the best preserved (Figures 2a and 3a), with the majority of the remains belonging to groups 1 and 2.

To quantitatively compare the preservation of the nauplii in each sediment, we used the ratio of the number of well‐preserved specimens (groups 1 and 2) to the number of poorly preserved ones (groups 3, 4, and 5) (Table 3). This ratio (we called it “degree of preservation”) varied widely across the sediments, being the highest in the montmorillonite and kaolinite and the lowest in the clinochlore. However, the score for the clinochlore was still higher than for the sediment‐free control (Table 3).

The results imply that the preservation potential of SBO improves considerably when the carcass is buried in sediment and that different sediments may provide different opportunities for preservation. In order to elucidate the main factors controlling the preservation, we analyzed changes in pH and mineral composition of the sediments and in the elemental and chemical composition of the carcasses.

#### 3.1.3 Decay‐induced changes of pH

The analysis revealed a complex pattern of pH gradients in the experimental tubes. Inside the sediments with the buried carcasses, the pH varied from acidic (pH 6.05 in the kaolinite) to alkaline (pH 11 in the montmorillonite) (Table 4). This is very different from the original (control) values of the artificial sea water (pH 7.8) which was used in experiments with the kaolinite, silica, clinochlore and chamosite, and fresh water (pH 7.0) used in the montmorillonite experiment. The pH of the liquid above the sediment with nauplii became more alkaline than inside the sediments (except montmorillonite). The difference between the pH in the middle of the sediment and the supernatant was more pronounced in the experimental tubes with nauplii than in the controls without nauplii (Table 4). Besides, in three experimental sediments (kaolinite, silica, and clinochlore), the pH had a local maximum in the water–sediment interface (Table 4, shown in bold). This means that the pH in the topmost layer of the sediment was higher than in the middle of the sediment and in the water column above the sediment (silica, clinochlore), or, in the case of the kaolinite, there was a thin layer of water immediately above the sediment where the pH was lower (7.04) than in the topmost sediment (7.13), although elsewhere in the water column the pH was high (7.76; Table 4).

**TABLE 4:** pH in the different layers of experimental sediments with *A. salina* nauplii (*Artemia*) and controls without nauplii

Sediment	Initial pH of water	Water column	Interface water/sediment	Sediment (middle part)
Montmorillonite + *Artemia*	7.0	9.63–9.70^b^	9.77–10.1^c^	10.9–11.0^d^
Kaolinite + *Artemia*	7.8	7.76	**7.04, 7.13** ^a^	6.05
Kaolinite control	7.8	7.5	7.7	7.9
Art. SiO_2_ + *Artemia*	7.8	8.20–8.25	**8.35–8.44**	7.78–7.80
Art. SiO_2_ control	7.8	7.71–7.81	**8.6**	7.70–7.76
Chamosite + *Artemia*	7.8	8.48	7.00–7.04	6.83–6.91
Chamosite control	7.8	8.05–8.07	7.4	7.09–7.11
Clinochlore + *Artemia*	7.8	8.7	**8.80–8.92**	8.17–8.22
Clinochlore control	7.4–7.8	7.1–7.4	6.22–6.45	5.9–7.3

Note

Local maxima at water/sediment interface are shown in bold. Each number represents the results of three measurements; intervals are shown if these results were different.

^a^ 7.04 immediately above the sediment, 7.13 in the topmost sediment.

^b^ Topmost part of the red layer.

^c^ Middle of the red layer.

^d^ 2 cm above the bottom (see Figure 1m)

This complex pattern of pH gradients becomes even more intricate when we consider the light‐colored spots around the carcasses. These spots probably imply lower local pH because the discoloration of the otherwise evenly darkened kaolinite or dark‐green clinochlore can be explained by the acidic dissolution of dark‐colored compounds (e.g., hydrotroilite in the kaolinite sediment (Naimark, Kalinina, Shokurov, Markov, et al., 2016)). With our equipment, we could not directly measure the pH within the spots because they are too small. However, microenvironmental pH minima have been shown to form locally around decaying biomass in marine sediments (Zhu et al., 2006).

The results are compatible with the idea that decaying organic matter induces chemical changes in the surrounding medium that potentially can affect the sediment and the set of ions released by sediment particles (see below).

#### 3.1.4 Biochemical composition of the carcasses: Spectral characterization

There are two main hypotheses that consider the biochemical components of the soft body as the key factor of preservation. The first one is focused on the presence of recalcitrant tissues such as chitin (Butler et al., 2015), while the second one emphasizes the stability of proteins tanned by aluminum ions (Naimark, Kalinina, Shokurov, Markov, et al., 2016; Wilson & Butterfield, 2014). To evaluate the importance of the two mechanisms, we assessed the sustainability of chitin and proteins in the carcasses with contrasting degrees of preservation (Figure 3a,b) using FT‐IR spectroscopy (see Section 2; Figure 3c–h).

In all carcasses (except for the sediment‐free control), the FT‐IR profiles consisted of two parts: the bands corresponding to chemical bonds in organic molecules, and the bands that correspond to the mineral (inorganic) components of the sediment. We first considered the organic part (Figure 3c–e).

**FIGURE 4:**
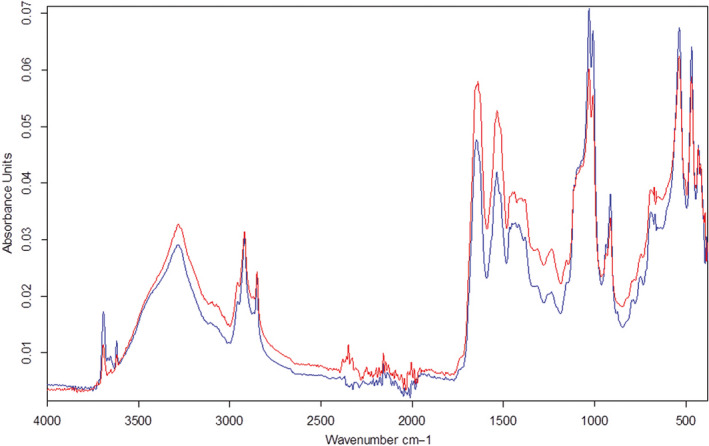
Biochemical composition of the carcasses: FT‐IR spectra for the best and worst preserved specimens from the kaolinite. Red line—preservational groups 1 and 2 (best preserved specimens), blue line—groups 4 and 5 (worst preserved specimens)

The spectra of all the studied *A. salina* samples from the different sediments were similar. The spectral pictures of the excellently preserved montmorillonite‐hosted specimens and poorly preserved clinochlore‐hosted samples showed little to no difference (Figure 3d,e). In the carcasses from different clays with different degree of preservation, the same peaks were present, and the differences affected only the intensities of the peaks. Moreover, the best and worst preserved specimens belonging to groups 1 and 4–5 from the kaolinite also had very similar FT‐IR spectra (Figure 4). The spectra also did not differ much from the two controls: live nauplii and the decayed sediment‐free control (Figure 3c). The protein characteristic bands (known as Amide I and II between 1,600 and 1,720 cm^−1^ and 1,510 and 1,580 cm^−1^ respectively; Figure 3e) varied little across the carcasses from different sediments. The same is true for the chitin characteristic bands (between 3,400 and 3,500 cm^−1^ and 2950–2800 cm^−1^ (Negrea et al., 2015); Figure 3d). The difference between nauplii from ASW and sediments seen in Figure 3e at 1560–1580 cm^−1^ can be attributed to conjugated systems like diketone, keto‐esters, and keto–enol structures. This difference is probably due to oxidation of organic matter which was higher in ASW than in the experimental sediments.

The second, inorganic part of the spectra occupies intervals at wavelength <1,300 cm^−1^. In all exhumed carcasses, these profiles repeated the spectra for the corresponding sediments (Figure 3f,g). In the SEM images, we could see small (<1 µm) sediment particles that stayed attached to the organic surface (Figure 2g) even after multiple intensive rinsings. So, can the similarity of the spectra of carcasses and sediments be explained by mere adherence of inorganic particles to the carcasses? Probably not, because the 1‐day control specimens, which had been kept in the kaolinite sediment for one day after death and then rinsed according to the common protocol, showed a much less pronounced signature of kaolinite (Figure 3h). This means that mineral particles did not adhere to a nauplial carcass immediately after deposition, but it took some time for the minerals to form strong mineral–organic bonds or to become incorporated into organic tissues.

Overall, the results presented in this section do not demonstrate any simple relationship between the rate of decomposition of chitin and/or proteins and the degree of preservation of the carcasses.

#### 3.1.5 Elemental composition of the carcasses

**FIGURE 5:**
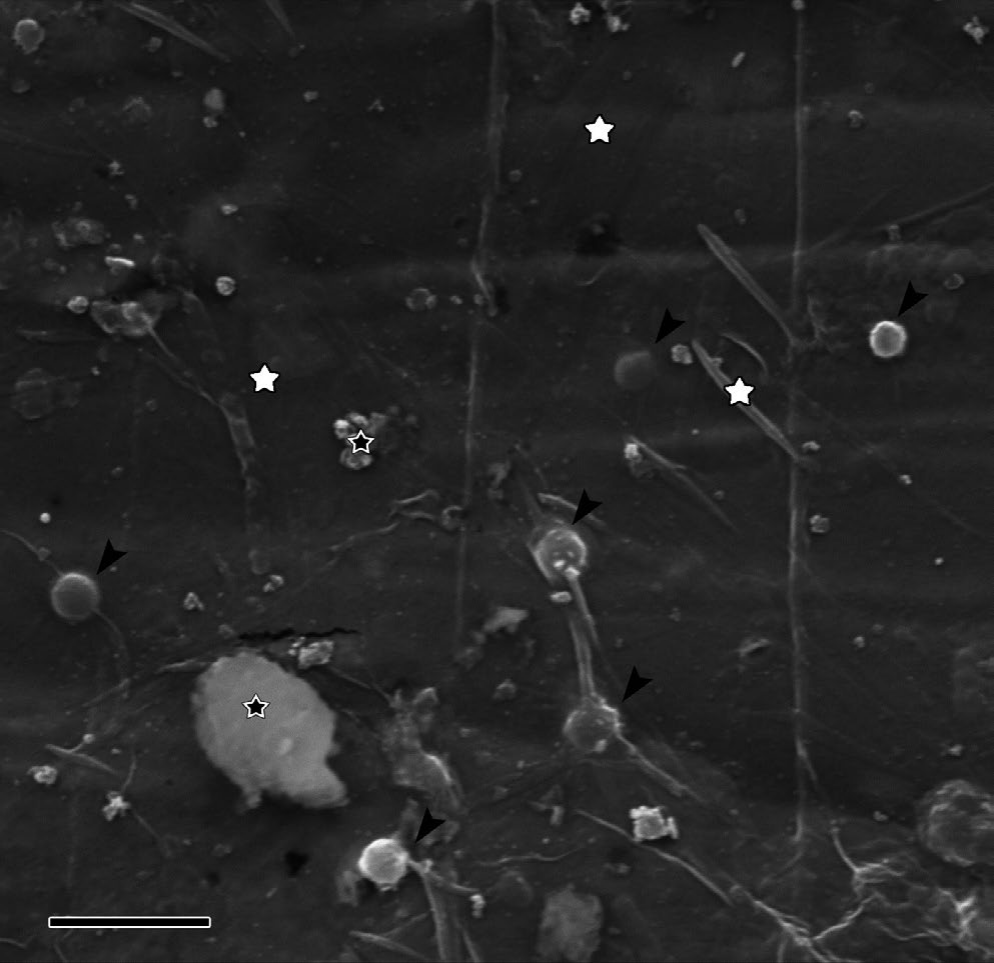
Example of a body surface fragment with points suitable for SEM/EDX analysis of the body (white asterisks) and sediment particles (black asterisks). The carcass is from the chamosite. Bacterial cocci are also visible (arrows). Scale bar 4 μm

In this analysis, we aimed to identify inorganic ions that enhance the preservation of SBO. Thus, we focused on the correlation between the deposition of different elements on the carcasses and the degree of their preservation. We used SEM/EDX multiple point analysis to measure chemical elements (a) in the patches of tissues free of visible sediment particles and bacteria and (b) in the sediment particles that were adhered to tissues (Figure 5 and Table 5).

**TABLE 5:** Elemental composition of the exhumed carcasses and sediment particles: SEM/EDX point analysis (at 15 keV, on copper support)

Sediment	Location	*n* (points)	CO_2_	MgO	SiO_2_	Al_2_O_3_	FeO	CaO	Cl
Montmorillonite	Body	10	43.8 (6.3)	0.2 (0.3)	5.3 (3.4)	1.8 (1.9)	0.1 (0.3)	0.2 (0.3)	0.6 (0.4)
	Particles	5	9.9 (1.3)	0.9 (0.1)	27.2 (1.1)	4.7 (0.4)	1.0 (0.3)	0.8 (0.1)	0.2 (0.1)
Kaolinite	Body	2	25.6 (0.8)	0 (0)	1.2 (0.7)	1.2 (0.7)	0 (0)	1.0 (0.3)	0.2 (0.3)
	Particles	2	21.6 (1.3)	0.1 (0.1)	4.6 (1.5)	4.4 (1.4)	0 (0)	1.6 (0.5)	0.4 (0.2)
Artificial silica	Body	14	25.6 (1.3)	0.2 (0.1)	1.5 (1.8)	0.04 (0.1)	0 (0)	0.9 (0.9)	1.4 (1.2)
	Particles	15	18.0 (3.6)	0.6 (0.3)	11.8 (6.6)	0.1 (0.1)	0 (0)	0.4 (0.3)	5.2 (2.5)
Chamosite	Body	10	68.9 (4.6)	0.03 (0.1)	0.2 (0.4)	0.2 (0.3)	1.4 (0.7)	1.1 (0.9)	0.1 (0.1)
	Particles	15	17.4 (4.2)	0.4 (0.1)	7.0 (2.1)	6.0 (1.8)	11.1 (3.4)	0.3 (0.2)	0.1 (0.2)
Clinochlore	Body	25	26.8 (0.4)	0.3 (0.3)	0.2 (0.2)	0.1 (0.2)	0 (0)	0.4 (0.3)	0 (0)
	Particles	9	14.8 (4.8)	10.2 (3.5)	6.5 (2.7)	5.7 (2.3)	2.4 (1.1)	0.7 (0.7)	0 (0)
Artificial sea water	Body	12	25.9 (2.1)	0.2 (0.6)	0 (0)	0 (0)	0 (0)	1.9 (3.2)	0.3 (0.5)

Note

Content of the elements is shown (mean (standard deviation); wt% as oxides) in the exhumed and rinsed nauplii (Body) and in the adhered sediment particles (Particles). See examples of the points in Figure 5.

**FIGURE 6:**
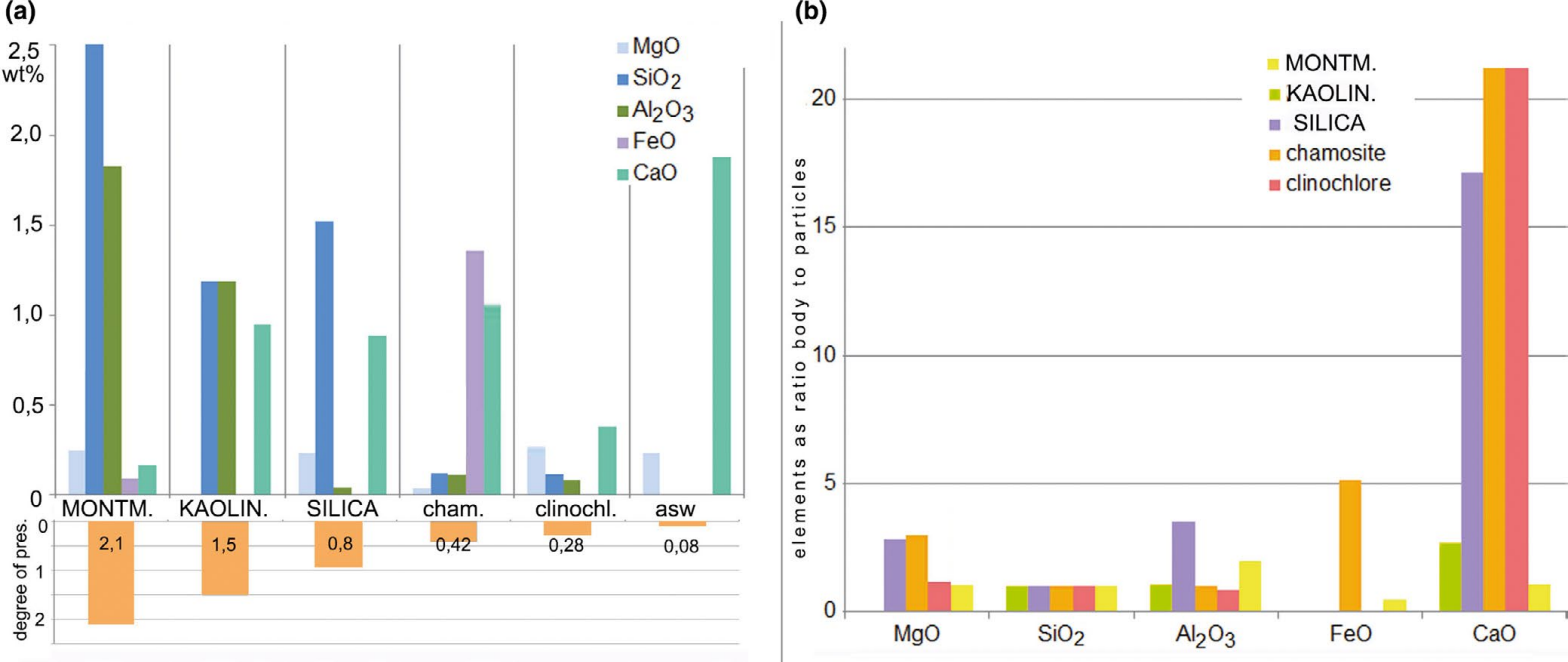
Elemental content of the body tissues and adhered sediment particles. Data from SEM/EDX analysis (Table 5). (a) Absolute elemental content (average weight percentage; see Table 5 for standard deviations) of the body tissues. Aluminum and silicon are increased in the carcasses from the montmorillonite, kaolinite and silica (the three sediments that ensure good preservation). The degree of preservation (Table 3) is shown on the lower diagram. Sediments with better preservation are depicted in capital letters on the horizontal axis. (b) Relative elemental content: body tissues relative to adhered sediment particles, normalized by silicon: ([Element in body]/[Silicon in body])/([Element in particles]/[Silicon in particles]). This ratio shows the extent to which the element enters the carcasses after being dissolved from the sediment

In the three sediments with the highest degree of preservation (montmorillonite, kaolinite, and silica, Table 3), the carcasses were enriched in aluminum and silicon. In the chamosite and clinochlore, where the preservation was poor, aluminum and silicon entered the tissues in minimal amounts (Table 5). Iron appeared in increased amounts in the well‐preserved carcasses from the montmorillonite and in the poorly preserved carcasses from the chamosite. The chamosite‐hosted carcasses contained much more iron and less magnesium than the carcasses from the clinochlore, in concordance with the initial chemical composition of the two sediments (Tables 2 and 5 and Figure 6a).

Calcium was detected in the carcasses from all sediments and ASW, regardless of the initial elemental composition of the sediments and water. The results imply that calcium was effectively absorbed by the carcasses both from the sediment and from the water (Figure 6b). Other elements from the dissolved sediments (Al, Si, Mg, Fe) apparently have entered the carcasses in a more passive way, and this is why the greater the amount of an element in the sediment, the higher is its content in the body tissues (Figure 6b).

**FIGURE 7:**
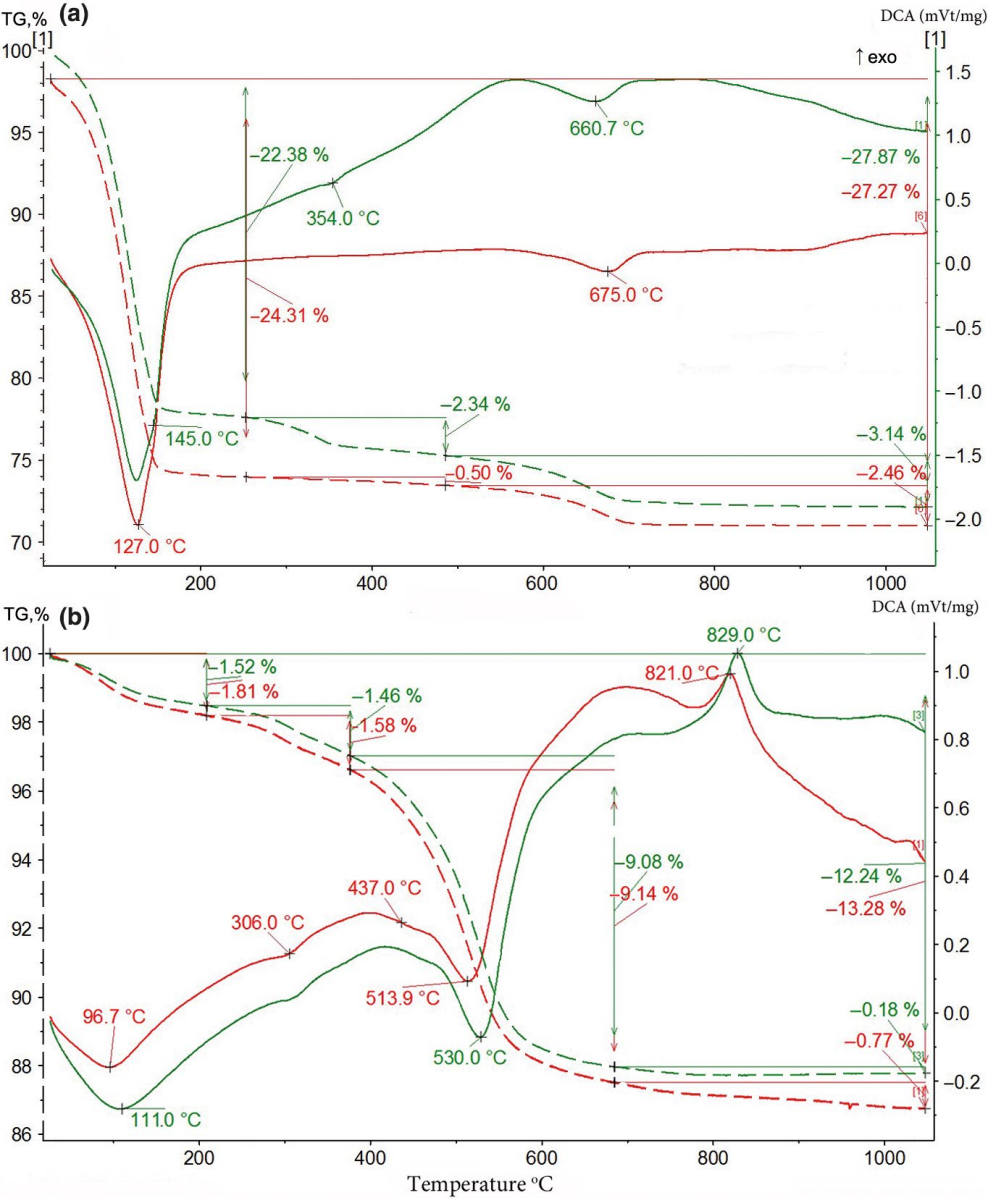
Results of Simultaneous Thermal Analysis: thermogravimetry (TG, dashed lines) and differential scanning calorimetry (DCA, solid lines; exo—exoeffect). (a) Montmorillonite, (b) chamosite. Green lines—experimental sediments, red lines—control sediments without *Artemia salina*. The changes in the sediments are manifested in the following features of the curves. New endoeffect at 354°C of ferrihydrite (Földvári, 2011) and shift in endoeffect from 675.0 to 660.7°C shown in A indicate structural disintegration of the montmorillonite. Shift of the first endoeffect from 96.7 to 105°C shown in B indicates accumulation of amorphous phase in the chamosite; shift of the second endoeffect from 513 to 530°C marks leaching of iron from the brucite layer; and higher exoeffect 829°C (in comparison with 813° in the control sediment) indicates disintegration of the talc layer of the chamosite

Different elements appeared in the solutions due to leaching of the corresponding sediments. This is in concordance with mineralogical transformations detected in the experimental sediments. The results of mineralogical analysis (STA, see Section 2) of the kaolinite and clinochlore were reported previously (Naimark, Boeva, et al., 2018; Naimark, Kalinina, Shokurov, et al., 2018). The results for the chamosite and montmorillonite are shown in Figure 7. They show that decaying organic matter promotes structural disintegration of the sediment and leaching of ions. In the montmorillonite, the strong alkaline environment accelerated the release of silicon species to a greater extent than in the artificial silica sediment with moderate alkalinity (Crundwell, 2014).

#### 3.1.6 Accumulation of iron

**FIGURE 8:**
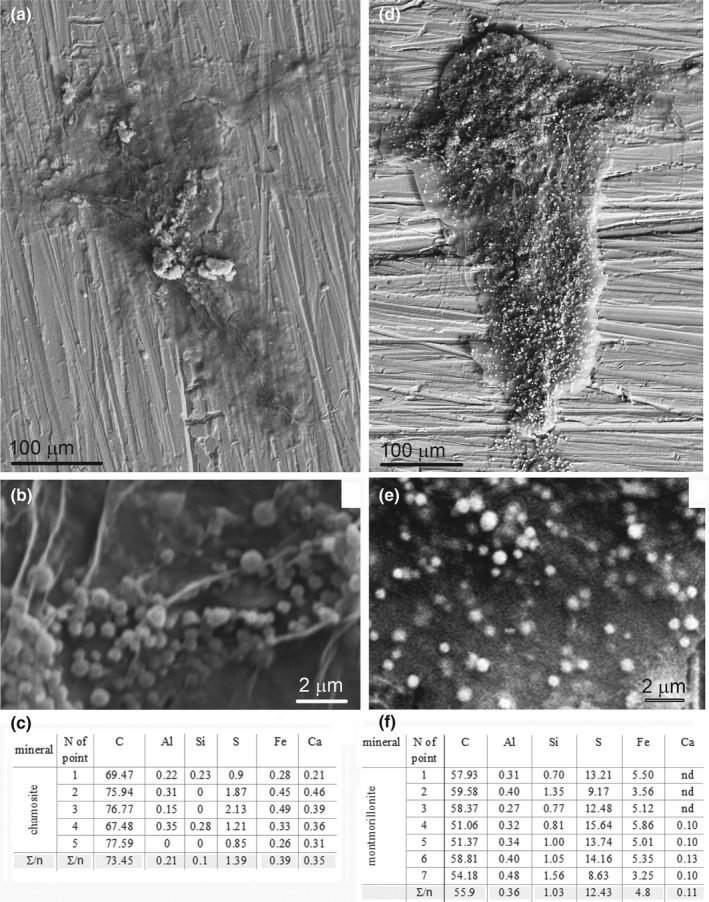
Samples from chamosite and montmorillonite with iron‐ and sulfur‐rich spherules. (a) Nauplius from the chamosite (on a copper support) with Fe‐S spherules. (b) Enlarged portion from (a) showing spherules. (d, e) The same from the montmorillonite. Note the spherules (whitish dots) in (d) scattered all over the surface. (c, f) Elemental content of the spherules (SEM/EDX analysis at 20 kEv, values are atomic percentages): (c) from the chamosite, (f) from the montmorillonite; Fe/S atomic ratio is about 1:3

The accumulation of iron in the buried carcasses needs additional clarification. SBO fossils are often pyritized to a varying extent, and the deposition of iron has been considered among the main mechanisms for SBO preservation (e.g., Schiffbauer et al., 2014). Two of our experimental sediments, chamosite and montmorillonite, showed interesting signatures of iron accumulation associated with the carcasses. Some exhumed carcasses from these sediments were scattered with small spherules either attached to the organic surface or located inside the thin integuments (Figure 8). These spherules varied widely in size, which is common for mineral particles but unusual for bacterial pseudomorphs (Schopf, 1976). According to the SEM/EDX point analyses (Figure 8c,f), the spherules contained Fe and S in an atomic ratio of approximately 1:3, which is consistent with ongoing pyritization (Rickard & Luther, 2007).

Mineralogical STA analysis confirmed that, in the presence of the buried nauplii, the montmorillonite disintegrated almost completely within 5 years (Figure 7a). The iron ions diffused from the mineral sediment upward along the pH and oxygen gradients and accumulated in the interfacial layer between the oxygen‐depleted and oxygenated phases. When concentrated in this layer, Fe^2+^ transformed to Fe^3+^ in a form of ferrihydrite, turning the coloration of this layer to brightly red (Figure 1m) and inducing a decrease of pH from 10.9–11.0 to 9.63–9.70 (Table 4). Ferrihydrite became stabilized by silicate ions that were released into the media due to the alkaline dissolution of montmorillonite (Rozalén et al., 2008).

Importantly, organic tissues served as a template for the nucleation of the released iron compounds. We failed to find such iron‐rich spheres in the loose sediment around the carcasses.

In the chamosite, where the initial iron content was high (Table 2) and iron subsequently leached from the mineral in the presence of the buried nauplii (Figure 7b), all iron‐rich spherules were also adhered to organic tissues (Figure 8). The results imply that such pyritization, even if it occurs rapidly, does not necessarily provide good preservation, because in our chamosite‐based system the preservation of *A. salina* was generally poor.

### 3.2 Deposition of aluminum on the surface of unicellular and multicellular organisms

**FIGURE 9:**
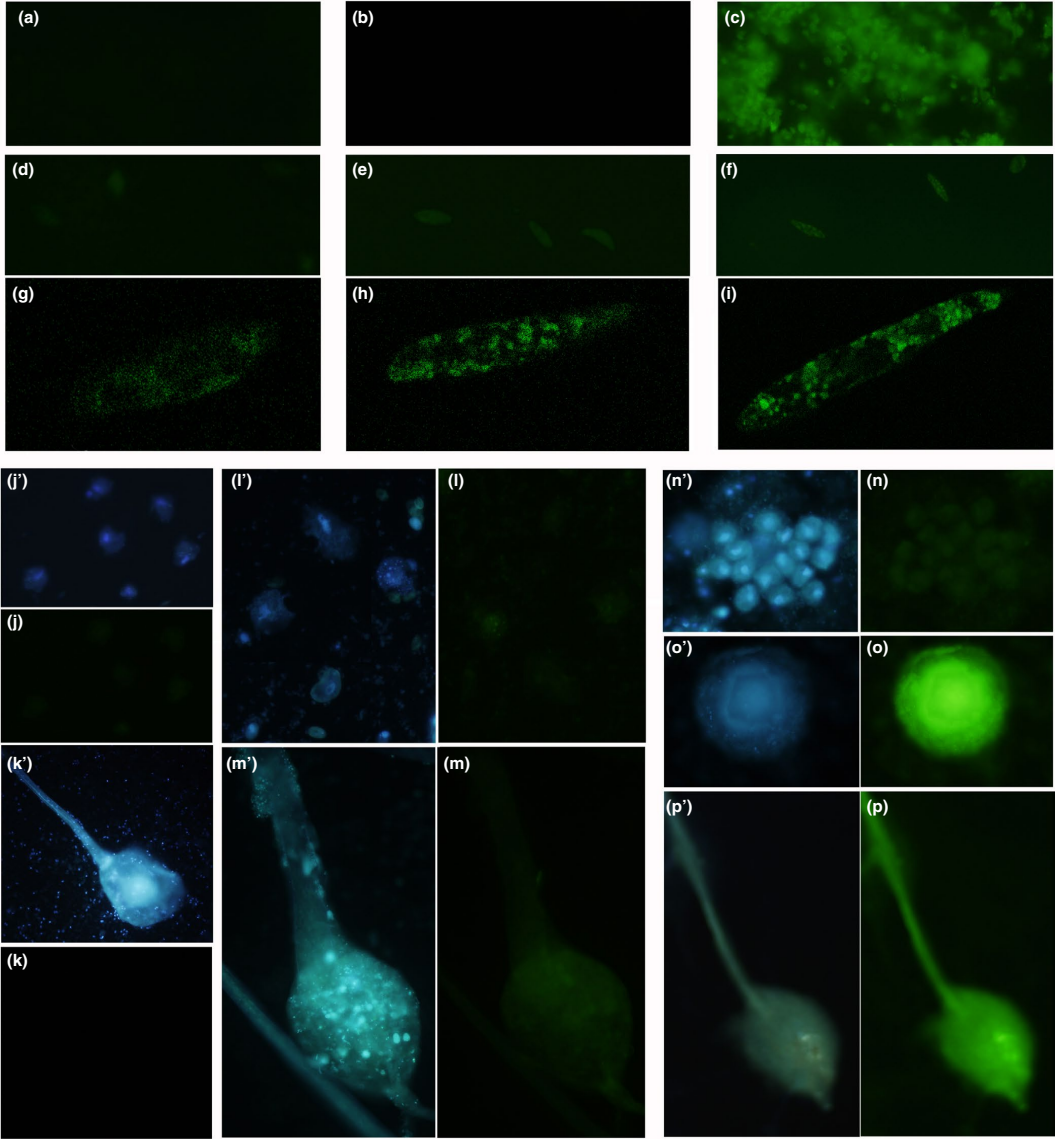
Deposition of aluminum on multicellular and unicellular organisms detected by fluorescent dye lumogallion (emission at 488 nm, green). (a–c) Sponge *Spongilla lacustris:* (a) autofluorescence, (b) lumogallion‐stained Al‐negative control, (c) lumogallion‐stained Al‐positive experiment. (d–i) flagellate *Euglena gracilis*: (d) autofluorescence, (e) Al‐negative; (f) Al‐positive; (g–i) the same under a confocal microscope to show intracellular localization of the fluorescence‐emitting organelles. (j–p) social ameba *Dictyostelium discoideum;* all samples were stained by cellular Hoechst dye to locate the cells; in each pair or images (e.g., j′, j), the first image (in this case, j′) shows fluorescence at 409 nm (Hoechst dye), while the second image (in this case, j) shows fluorescence at 488 nm (lumogallion). (j, l, n) Unicellular amoebae; (j) “autofluorescence” (Al‐negative, no lumogallion staining), (l) lumogallion‐stained Al‐negative control, (n) lumogallion‐stained Al‐positive experiment. (k, m, p) multicellular fruiting body; (k) “autofluorescence,” (m) lumogallion‐stained Al‐negative control, (p) lumogallion‐stained Al‐positive experiment. (o) multicellular culminant, lumogallion‐stained Al‐positive experiment. Fluorescence is bright green only in Al‐positive multicellular stages (o and p), implying that only multicellular stages absorb aluminum from the medium. Scale bars c–f, k, m, o, p—100 µm, g–i, j, l, n—20 µm

The sponge showed a very clear response to incubation with Al^3+^ and subsequent lumogallion staining. Autofluorescence in the nonstained control was negligible, as well as the fluorescence in the lumogallion‐stained Al‐negative control. However, Al‐incubated lumogallion‐stained specimens emitted bright green fluorescence (Figure 9a–c).

The cells of *E. gracilis* had moderate autofluorescence and relatively intense fluorescence in the Al‐negative control. The incubation with aluminum did not add any intensiveness to the emission as observed under the confocal microscope (Figure 9d–f). The confocal microscopy also allowed us to recognize that autofluorescence and emission in the Al‐negative control were mostly localized in some intracellular structures (probably chloroplasts and vacuoles), but not on or near the cell surface (Figure 9g–i). Importantly, in the cells incubated with Al^3+^, fluorescence of the outer pellicle was not more intense than in the Al‐negative cells (in fact, both fluorescent signals were equally low). The results imply that aluminum did not accumulate on the dead cells of *E. gracilis* in the same fast and efficient way as on the cells of the sponge.

The cells of *D. discoideum* at multicellular stages (culminants and fruiting bodies) emitted brightly at 488 nm (Figure 9o,p). The strong Al‐lumogallion signal from the multicellular structures was clearly different from the weak autofluorescence signal of the fruiting body (Figure 9k) and the weak emission of the Al‐negative control (Figure 9m). Single amoebae showed very low fluorescence in all cases (Figure 9j,l,n).

## 4 DISCUSSION

### 4.1 Decay‐induced chemical processes in the sediment

Decay in the sediment produces a range of chemical reactions that can enhance the preservation potential of SBO. A necessary prerequisite for many of them to start is the fine‐grained texture of the sediment that slows the diffusion of oxygen and dissolved ions (Allison, 1988). Clay particles are usually of fine and ultrafine size, and therefore, clays are expected to enhance the preservation potential of a buried carcass. Other sediments such as fine‐grain silica in our experiments can also trigger reactions necessary for preservation.

The arrested diffusion results in increasing concentrations of different ions around the decaying body. Chemical gradients thus established can form a spot‐like or layer‐like morphology in the sediment (Figure 1). Although this chemical heterogeneity has been often overlooked in the discussion of SBO preservation, it invokes at least two conditions that can favor preservation. The first one is the fast accumulation of mineralizing agents (e.g., ions of Ca, Mg, Al, Si, Fe, P) released via acidic or alkaline hydrolysis of the sediment. The second one is the peak of the pH at the water/sediment interface (Table 4). Similar peaks can be found in the topmost layers of some modern organic‐rich marine sediments (Zhu et al., 2006). If there is no bioturbation, this peak of the pH can sometimes induce the formation of calcium‐rich sealing cement at the water/sediment interface (Naimark, Kalinina, Shokurov, Markov, et al., 2016). Surface cementation of this kind is a widely recognized phenomenon in SBO fossil localities, although its origin is poorly understood (Gaines et al., 2012). Our results suggest that it may result from the increased pH in the top layer of organic‐rich sediment.

The repertoire of mineralizing ions depends on the type of sediment. Its chemical and physical properties determine how the pH will change in response to organic decay, and which elements will leach. We found a good concordance between the transformation of the sediments and the composition of cations permeating the carcasses. Therefore, the diversity of primary host sediments is expected to produce a diversity of the resulting elemental content of SBO fossils.

Five experimental sediments led to quite different degrees of preservation of the model soft‐bodied organism (Table 3). We used them as models and made no attempt to mimic natural deposits. Thus, it does not necessarily mean that the same sediments would provide the same degrees of preservation in natural environments. Their potential to preserve soft carcasses depends on other factors as well. The montmorillonite experiment is revealing in this sense. It is known that soft‐bodied fossils are rare in rocks containing montmorillonite or its hydrated product illite (Anderson et al., 2018), apparently because montmorillonite is comparatively stable in the common burial conditions in nature. In our experimental conditions, however, the pH in the montmorillonite sediment became alkaline (Table 4), thus triggering the dissolution of the montmorillonite and mineralization and preservation of the buried soft‐bodied organisms.

More research is needed to elucidate the particular factors mediating the decay and preservation of SBO in different sediments. We acknowledge that, due to the limited coverage of environments and model organisms in our experiments, they probably do not cover the entire range of conditions suitable for SBO fossilization. Moreover, there are general limitations to such experiments, because even when morphology is preserved for 5 years, it is difficult to say how it would look after 100 million years. However, our results imply that different fine‐grained sediments (such as the clays and the artificial silica we used) can, under some conditions, facilitate the formation of specific microenvironments and chemical gradients in the vicinity of the buried carcasses that can affect their preservation potential in a variety of ways.

### 4.2 Deposition of aluminum and silicon is an important mechanism of preservation of the soft‐bodied organisms

The ions released from the mineral matrices exhibit different preservation ability. Aluminum and silicon ions (released in the form of amphoteric oxides) provided the best preservation of the organic structures (Figure 6). We acknowledge that some other elements, which were absent or rare in our experimental context, may also enhance preservation (e.g., phosphorus).

Magnesium, iron, and calcium were readily deposited on the organic tissues, but their abundance in the carcasses was not associated with better preservation, indicating that these elements probably are not very effective in preventing decay. Nevertheless, owing to their fast deposition on the carcasses, they probably can create 2D‐organo‐mineral “portraits” in the sediment that are visually accentuated by the high concentration of the corresponding element (especially iron). In the presence of aluminum and/or silicon that slow down decay, carbonaceous films veneered by iron, calcium, or magnesium may be formed on the surface of the carcasses. In the case of iron‐rich sediments, a pyritized veneer can penetrate inside the tissues to produce a more or less pyritized fossil (Schiffbauer et al., 2014). It needs to be emphasized that the fast deposition of an element on the carcass and its subsequent accumulation in decaying tissues does not necessarily mean that this element enhances preservation. We need to distinguish the two processes: the fast deposition of elements and their action as preservatives. This is especially important in the context of the current debates about the role of iron in preservational pathways (Newman et al., 2019; Schiffbauer et al., 2014). Iron is very common in sedimentary rocks, and so it is expected to be frequently found in SBO fossils (Petrovich, 2001); however, this does not mean that iron was essential for their preservation.

In all experiments reported here, including the sediment‐free control, calcium ions entered the body tissues very effectively (Figure 6 and Table 5). Thus, some SBO fossils from aluminum‐ and silicon‐containing sediments can be expected to be high in calcium, which seems to be consistent with the data on elemental contents of fossils from clays (Orr et al., 1998). However, without aluminum and silicon (or other putative preservation‐enhancing ions), calcium does not appear to ensure good preservation. Interestingly, even highly calcified SBO fossils were found coated with a thin layer of aluminosilicate, for example, in the Silurian Herefordshire Lagerstätte (Siveter et al., 2020).

In our experiments, good preservation was associated with strong adherence of sediment particles to decaying bodies; some specimens were wrapped in clay “envelopes” (Figure 2). As a result, surface body tissues became more rigid and the body retained its shape (the characteristic feature of the preservational groups 1 and 2).

Importantly, the chemical pathways of SBO preservation discussed here do not require any unique environmental conditions and occur relatively quickly. Preservational processes take place whenever a sufficient amount of dead organic bodies is buried under a layer of fine‐grained sediment. This probably requires catastrophic burial, as has been suggested for many different Lagerstätten (reviewed in Naimark, Kalinina, Shokurov, Markov, et al., 2016). According to the mineral composition of the sediment and probably some other characteristics of the system, buried carcasses may become mineralized and fossilized in different modes and by different compounds. Aluminum and silicon are possibly required for SBO preservation, but they are among the most abundant elements in sedimentary rocks. Owing to diverse and ubiquitous contexts for SBO preservation, Konservat‐Lagerstätten localities are expected to be quite numerous. The recent data seem to confirm that SBO fossil localities are not taphonomic rarities: During the last two decades, ten times more Lagerstätten have been described than during more than 100 years since their discovery in the 19th century: from 53 Lagerstätten occurrences in 1997 to 670 in 2017 (Muscente et al., 2017).

### 4.3 Cell adhesion molecules probably play a role in preservation of the soft‐bodied organisms

Despite the popular idea that durable integuments (e.g., chitinous cuticles) are essential for fossil preservation, in some Lagerstätten fossil animals with and without such durable tissues are found in comparable numbers (Fu et al., 2019).

The results of our FT‐IR analysis (Figure 3) show that differently preserved specimens have similar spectral portraits, implying that different biochemical components probably had similar rates of decay. More specifically, we found that the best preserved specimens were not those that retained more chitin or proteins. Although we were not able to measure these components precisely, and FT‐IR analysis may be not sensitive enough to capture the differences, the similarity of the spectra probably indicates that morphological preservation may be somehow independent of the preservation of tissue structure and composition, and depend more on the general rate of decay. We thus tentatively conclude that soft‐bodied Metazoa with different bulk biochemistry may have comparable chances of fossilization and that durable chitinous integuments may play only a secondary role in the preservation of SBO.

This conclusion seems to contradict previously published data showing that different tissues decay at different rates (e.g., Sansom, 2014; Sansom et al., 2010). However, all these earlier findings were based on decay experiments in water, whereas our study is focused on decay in fine‐grain sediment, which is a very different process.

Given that Konservat‐Lagerstätten are diverse and numerous and that durable tissues are not an imperative for SBO preservation, then some other biochemical factors may be expected to play the key role in different preservation pathways. The idea of key biochemical factors seems plausible because the mineralization of a dead organism probably should begin with binding of mineral ions to some cell surface molecules. The obvious candidates are acidic and very acidic proteins, glycoproteins and polysaccharides which have been shown to facilitate nucleation of mineral crystals during skeletal biomineralization in eukaryotes (Marin & Luquet, 2007). We hypothesize that there is a special group of these acidic biomolecules that play a crucial role in the fossilization of SBO: cell adhesion molecules and related extracellular matrix molecules (CAMs and ECMs) that evolved in multicellular animals (and some other organisms like social amoebae) to ensure cell–cell and cell–substrate adhesion.

CAMs and ECMs have the ability to adhere to mineral particles and bind ions; many types of CAMs, such as cadherins and integrins, are known to bind inorganic ions as part of their physiological function (Brown et al., 2018; Sotomayor & Schulten, 2008). For example, the physiological function of integrins and cadherins depends on their ability to bind metal cations, and their extracellular domains contain multiple Ca^2+^ binding sites (Albelda & Buck, 1990; Sotomayor & Schulten, 2008) which probably can nonspecifically bind other cations as well (Petukh & Alexov, 2014). Another important type of CAMs capable of nonspecific binding of cations is mucin glycoproteins which contain negatively charged sulfated glycans, sialic acid and multiple oligosaccharide residues (Marczynski et al., 2020).

From this perspective, the results of our experiments with deposition of aluminum seem logical. They demonstrated that the multicellular sponge deposited Al on the surface of its cells while single‐celled *Euglena* did not. This correlates with a large set of CAMs and ECMs in sponges and their absence in Euglenozoa (Fahey & Degnan, 2010; Seymour et al., 2004). Conversely, multicellular developmental stages of *D. discoideum* deposited Al, while unicellular amoebae did not. This is probably because the multicellular stages of *D. discoideum* express some aluminum‐binding molecules which are present on the cell surface and retain their affinity to aluminum after cell death; these molecules are absent at the unicellular stage. In the case of social amoebae, the most plausible candidates are indeed cell adhesion molecules and related extracellular molecules (CAMs and ECMs). Unicellular stages (single amoebae) do not express CAMs, while the progression of *D. discoideum* through its multicellular stages is accompanied by the expression of several cell surface CAMs (Coates & Harwood, 2001; Siu et al., 2011).

Given the presence of CAMs and ECMs in multicellular organisms, the fossilization of their soft carcasses via fast binding of certain inorganic ions (e.g., aluminum and silicon ions) would be an inevitable consequence of a certain stage of evolution of multicellularity, when a set of CAMs and ECMs became sufficiently developed (Abedin & King, 2010). If our hypothesis is correct, then the crown groups of metazoans with fully developed sets of CAMs and ECMs would have a higher chance of being preserved in the fossil record compared to basal groups with less advanced or numerous CAMs and ECMs. For example, sets of adhesive molecules are poor or lacking in basal holozoans (including choanoflagellates), which, in the absence of a mineral skeleton, makes their fossilization very unlikely. We acknowledge that many more experiments with single‐celled holozoans and other protists are needed to verify our hypothesis.

The hypothesis explains several enigmatic features of the fossil record: the extreme scarcity of the record of basal Metazoa who probably had only a few primitive CAMs and ECMs; the poor record of nonskeletal holozoans which, according to phylogenetic reconstructions, should have been present since the Proterozoic and throughout the Phanerozoic (Betts et al., 2018; Dohrmann & Wörheide, 2017; Sharpe et al., 2015), and probably had no or very few CAMs (Abedin & King, 2010). The rich fossil record of bacterial mats can be explained by their sheaths of acidic polysaccharides and mucins (one class of CAMs).

Speculating on the role of CAMs and ECMs in preservation, we need to ask an important question. According to molecular phylogenies, the first multicellular animals must have appeared long before the Cambrian Explosion, probably as early as 740–800 Ma (Budd & Mann, 2020; Dohrmann & Wörheide, 2017). Why there is no rich fossil record of these early animals? The current explanation is that the earliest metazoans were so small and so unlike the later animals, that their fossils, even if found, are not likely to be correctly interpreted as animal fossils (Erwin et al., 2011). We hypothesize that, even more importantly, these basal metazoans had low fossilization potential because their CAMs were few and probably not very effective in providing strong cell–substrate and cell–cell contacts. It is possible that underdeveloped CAMs can decrease the preservation potential in two ways: first, by poor binding of preservation‐enhancing metal ions; second, by higher risk of disintegration of the organism after death. Apparently, much more experimental work is needed to clarify these points.

It is also worth to note that cell surface biochemistry and extracellular structures (e.g., cell walls) in plants, fungi, and many other eukaryotic clades differ from those in animals. Thus, their preservation is probably governed by other molecular mechanisms and should be addressed separately. This applies to the majority of the Proterozoic unicellular eukaryotic fossils (including various cysts, smooth walled and ornamented acritarchs) reviewed by Cohen and Macdonald (2015).

A critical review of the evolution of CAMs and ECMs and its comparison with the trends in the fossil record of Metazoa may bring interesting insights into the chronology of the early evolution of animals. The fast evolution and diversification of CAMs and ECMs probably can, to some extent, account for the explosive emergence of diverse multicellular animals in the Cambrian fossil record.

## CONFLICT OF INTEREST

The authors declare that they have no competing interests.

## AUTHOR CONTRIBUTION

**Elena Naimark:** Conceptualization (lead); Formal analysis (lead); Investigation (lead); Methodology (equal); Supervision (lead); Visualization (lead); Writing‐original draft (lead); Writing‐review & editing (equal). **Dmitry Kirpotin:** Conceptualization (equal); Formal analysis (supporting); Methodology (equal); Writing‐original draft (equal); Writing‐review & editing (equal). **Natalia Boeva:** Formal analysis (equal); Investigation (equal); Methodology (supporting); Writing‐original draft (supporting); Writing‐review & editing (supporting). **Vladimir Gmoshinskiy:** Investigation (equal); Methodology (supporting); Writing‐original draft (supporting); Writing‐review & editing (supporting). **Maria Kalinina:** Conceptualization (equal); Formal analysis (equal); Writing‐original draft (equal); Writing‐review & editing (supporting). **Yulia Lyupina:** Formal analysis (equal); Investigation (equal); Methodology (equal). **Alexander Markov:** Conceptualization (equal); Formal analysis (equal); Writing‐original draft (equal); Writing‐review & editing (equal). **Michail Nikitin:** Formal analysis (supporting); Investigation (supporting); Methodology (equal); Writing‐original draft (supporting); Writing‐review & editing (supporting). **Alexander Shokurov:** Formal analysis (equal); Investigation (equal); Methodology (supporting); Writing‐original draft (supporting); Writing‐review & editing (supporting). **Dmitry Volkov:** Formal analysis (equal); Investigation (equal); Methodology (supporting); Writing‐original draft (supporting); Writing‐review & editing (supporting).

## Data Availability

Data supporting the results in the paper are available at Dryad, https://doi.org/10.5061/dryad.gb5mkkwnt (proportions of preservational groups in different sediments, elemental composition of the exhumed carcasses and sediment particles, pH in the different layers of experimental sediments).
